# Discharge Against Medical Advice in Cancer Patients: Insights from a Multicenter Study in Germany

**DOI:** 10.3390/cancers17010056

**Published:** 2024-12-28

**Authors:** Sarah Krieg, Sven H. Loosen, Christoph Roderburg, Andreas Krieg, Karel Kostev

**Affiliations:** 1 Department of Inclusive Medicine, University Hospital Ostwestfalen-Lippe, Bielefeld University, 33617 Bielefeld, Germany; sarah.krieg@mara.de; 2 Department of Gastroenterology, Hepatology and Infectious Diseases, University Hospital Duesseldorf, Medical Faculty of Heinrich Heine University Duesseldorf, 40225 Duesseldorf, Germany; sven.loosen@med.uni-duesseldorf.de (S.H.L.); christoph.roderburg@med.uni-duesseldorf.de (C.R.); 3 Department of General and Visceral Surgery, Thoracic Surgery and Proctology, University Hospital Herford, Medical Campus OWL, Ruhr University Bochum, 32049 Herford, Germany; 4 Epidemiology, IQVIA, 60549 Frankfurt, Germany; karel.kostev@iqvia.com

**Keywords:** discharge against medical advice (DAMA), cancer, risk factors, epidemiology, health behavior, Germany

## Abstract

Background: Discharge against medical advice (DAMA) disrupts continuity of care and is associated with increased readmission rates, morbidity, and mortality. While extensively studied in general hospital populations, its prevalence and associated factors in cancer patients, where treatment adherence is critical for outcomes, remain underexplored. Methods: This multicenter, cross-sectional study analyzed anonymized data from the IQVIA hospital database, including cancer patients hospitalized in 36 German hospitals between January 2019 and December 2023. Multivariate logistic regression assessed associations between DAMA and factors such as age, sex, cancer type, metastases, and comorbidities. Results: Among 51,505 cancer patients, DAMA occurred in 0.9% of hospitalizations. The highest rates were observed in cancers of the lip, oral cavity, and pharynx (2.1%), larynx (2.0%), and liver (1.8%). DAMA was more frequent in younger patients (≤50 years) (OR: 1.73; 95% CI: 1.30–2.14) and males (OR: 1.46; 95% CI: 1.23–1.72). Distant metastases showed no significant association (OR: 0.96; 95% CI: 0.81–1.13). Conclusions: The findings suggest that DAMA in cancer patients is more strongly associated with demographic and social factors than with disease severity. These results provide a basis for exploring strategies that address underlying psychosocial and economic challenges during hospitalization, particularly in younger and male patients. Further research is needed to better understand these associations and their implications for clinical practice.

## Simple Summary

Discharge against medical advice (DAMA) is a complex phenomenon with the potential to disrupt care in hospitalized patients, particularly in oncology, where continuous treatment is crucial for managing disease progression and improving outcomes. This study aimed to explore the prevalence of DAMA among cancer patients in Germany and identify factors associated with its occurrence. Analysis of data from over 51,000 patients hospitalized across 36 facilities revealed a DAMA rate of 0.9%. Younger age (especially under 60 years) and male sex were associated with higher DAMA rates. Patients with cancers of the lip, oral cavity, pharynx, larynx, and liver had the highest DAMA prevalence, while distant metastases showed no significant association. These findings highlight the multifaceted nature of DAMA and suggest that non-clinical factors, such as psychological, social, and economic pressures, may play a critical role in patients’ decisions. This study provides a foundation for further research into strategies to better understand and address DAMA in vulnerable groups, improving care and outcomes for hospitalized cancer patients.

## 1. Introduction

Discharge against medical advice (DAMA), defined as a patient’s decision to leave the hospital before the treating physician recommends discharge, poses a significant challenge in healthcare. This phenomenon is associated with negative outcomes, including increased morbidity, mortality, and healthcare costs [1,2]. Patients who leave the hospital prematurely often do not receive adequate or complete treatment for their medical condition, which can ultimately lead to serious complications requiring readmission [3].

In a small prospective study involving 97 hospitalized patients, Hwang et al. investigated the rates and factors associated with readmission among patients who left the hospital against medical advice [3]. The findings showed that these patients had a significantly higher risk of readmission within 15 days compared with those discharged as recommended (21% vs. 3%; *p* < 0.001). Male sex and a history of alcohol abuse were identified as key predictors, emphasizing the role of behavioral and social factors [3]. Similarly, in a retrospective study, Fiscella et al. analyzed data from 1079 patients who had been admitted with acute myocardial infarction and, subsequently, DAMA [4]. The authors demonstrated that these patients were less likely to undergo revascularization procedures and exhibited a 40% increased risk of mortality or readmission due to myocardial infarction or unstable angina within two years of discharge [4].

Furthermore, DAMA presents treating physicians with a significant ethical dilemma. Physicians must reconcile the competing demands of respecting the patient’s autonomy and their professional obligation to ensure the patient’s well-being and provide appropriate medical care [5]. This challenge becomes even more pronounced when patients’ decisions are driven by factors such as psychosocial or economic pressures, raising questions about informed decision making and equitable care.

Although DAMA has been explored in various medical conditions, evidence focusing specifically on cancer patients remains limited [6,7,8,9,10]. Cancer care typically involves prolonged treatment regimens and high levels of patient engagement, making DAMA particularly concerning in this population. The complexity and severity of cancer may introduce unique challenges in managing DAMA, as factors such as treatment burden, psychological distress, and socioeconomic constraints could all contribute to a patient’s decision to leave prematurely. Given the potential consequences of DAMA in oncology, such as delayed treatment, disease progression, and poorer overall outcomes, understanding its determinants is essential for improving care delivery.

The present study aimed to address this gap by examining the prevalence and determinants of DAMA in cancer patients in a large, multicenter setting. Specifically, we analyzed the associations between DAMA and demographic and clinical variables, providing insights that could inform strategies to reduce DAMA and improve care adherence in this vulnerable population.

## 2. Materials and Methods

### 2.1. Data Source

This multicenter cross-sectional study was conducted in accordance with the STROBE (Strengthening the Reporting of Observational Studies in Epidemiology) guidelines [11], and a detailed STROBE checklist is provided as Supplementary Material. Data were derived from the IQVIA hospital database, which encompasses records from 36 hospitals in Germany, including specialized facilities, primary care centers, maximum and standard care hospitals, and university hospitals.

The §21 dataset follows a standardized format mandated by §21 KHEntgG (Krankenhausentgeltgesetz; Hospital Payment Act) and is transmitted by hospitals to the Institut für das Entgeltsystem im Krankenhaus (InEK; the Institute for the Payment System in Hospitals). To ensure compliance with data protection regulations, all export files are anonymized prior to transmission, removing identifiers such as patient and case numbers. The IQVIA database has been extensively utilized in previous epidemiological research, underscoring its reliability and relevance for public health studies [12,13].

### 2.2. Study Population and Outcome

**Figure 1:**
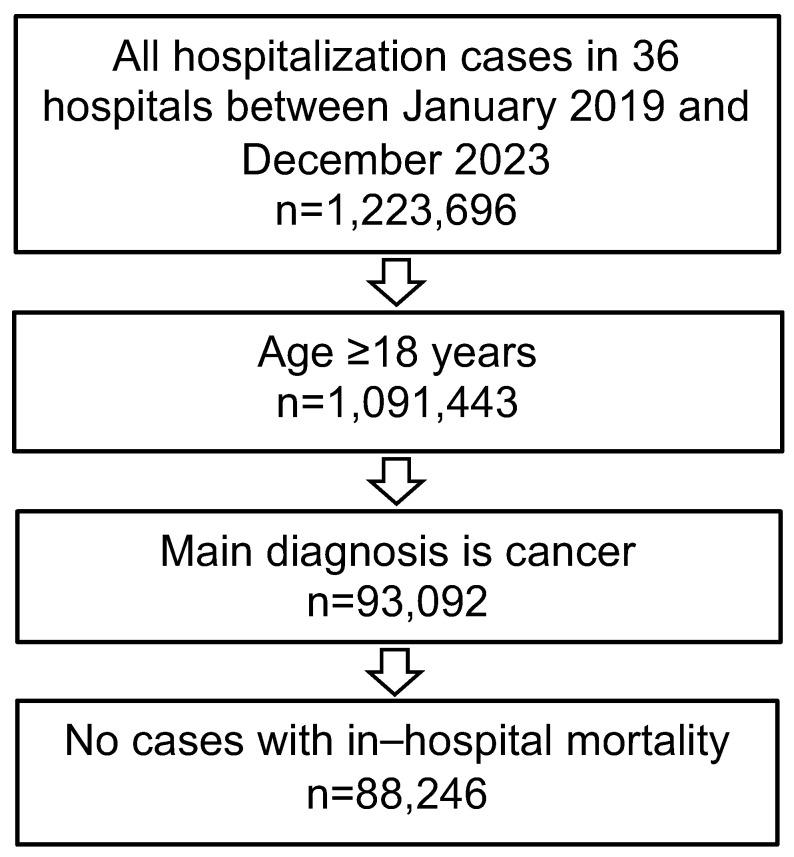
Selection of the study sample.

This study encompassed all hospitalizations of patients aged 18 years and older who received a primary diagnosis of cancer (ICD-10: C00–C97) during the timeframe of January 2019 to December 2023. Cases where in-hospital mortality occurred were excluded from the analysis (Figure 1). The primary focus of this study was to assess the prevalence of DAMA. DAMA rates were calculated for the entire cohort as well as for specific subgroups, including five age categories (≤50, 51–60, 61–70, 71–80, and >80 years), sex (women and men), presence or absence of distant metastases, and the most commonly reported cancer types. These cancer types included lip, oral cavity, and pharynx (ICD-10: C00–C14), esophagus and stomach (ICD-10: C15, C16), colon and rectum (ICD-10: C18–C20), liver (ICD-10: C22), pancreas (ICD-10: C25), larynx (ICD-10: C32), bronchus and lung (ICD-10: C34), skin (ICD-10: C43, C44), female breast (ICD-10: C50), female genital organs (ICD-10: C51–C58), prostate (ICD-10: C61), urinary tract (ICD-10: C64–C68), lymphomas (ICD-10: C81–C90), and leukemias (ICD-10: C91–C95).

### 2.3. Statistical Analyses

Multivariable logistic regression analyses were performed to examine the associations between demographic and clinical factors and DAMA. The dependent variable in these models was DAMA (yes/no), while the independent variables included age groups, sex, presence of metastases, cancer types, and secondary diagnoses observed in at least 5% of the study population. The secondary diagnoses included diabetes mellitus (ICD-10: E10–E14), lipid metabolism disorders (ICD-10: E78), hypertension (ICD-10: I10), coronary heart disease (ICD-10: I25), atrial fibrillation and flutter (ICD-10: I48), chronic kidney disease (ICD-10: N18, N19), and thyroid gland disorders (ICD-10: E00–E07).

Urinary tract cancer was selected as the reference group for logistic regression, as it is relatively common and occurs in both sexes. The results of the regression analyses were presented as odds ratios (ORs) with corresponding *p*-values, where *p*-values below 0.05 were considered statistically significant. All statistical computations were conducted using SAS version 9.4 (SAS Institute, Cary, NC, USA).

## 3. Results

### 3.1. Baseline Characteristics

A total of 88,246 hospitalization cases (51,505 cancer patients) were included in this study. The mean age was 66.5 (SD: 13.8) years, and 53.6% were female. The median length of stay in hospital was 4 days. The most frequent cancer type was female breast (12.4%), followed by bronchus and lung (10.8%) and colorectal cancer (9.8%). Hypertension (42.4%), diabetes mellitus (15.3%), and thyroid gland disorders (11.7%) were the most frequent co-diagnoses (Table 1).

### 3.2. Prevalence of Discharge Against Medical Advice

**Figure 2:**
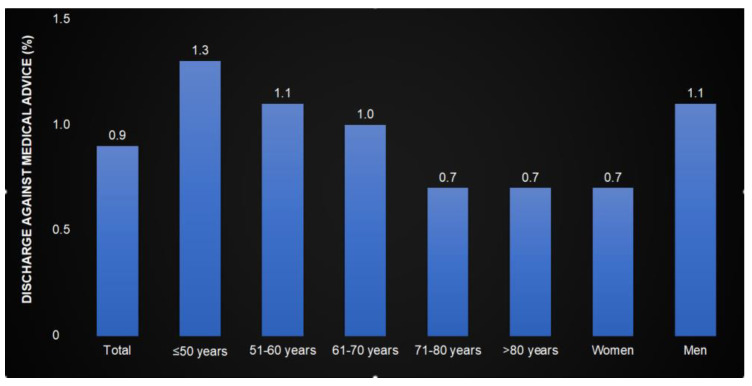
Discharge against medical advice in patients hospitalized for cancer by age and sex.

Of 88,246 hospitalization cases, 814 (0.9%) were registered as DAMA. The DAMA rate decreased from 1.3% in the age group of ≤50 years to 0.7% in the age group of >80 years and was higher among men (1.1%) than among women (0.7%) (Figure 2).

**Figure 3:**
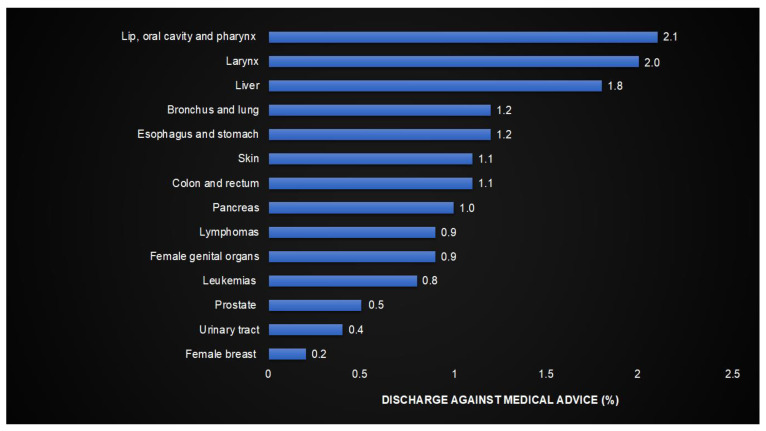
Discharge against medical advice in patients hospitalized for cancer by cancer type.

The highest DAMA rate was observed in patients with lip, oral cavity, and pharynx cancer (2.1%), followed by larynx (2.0%) and liver cancer (1.8%). The lowest DAMA rates were in patients with female breast cancer (0.2%), urinary tract cancer (0.4%), and prostate cancer (0.5%) (Figure 3).

Additionally, we analyzed DAMA patterns in patients with multiple hospitalizations. Among 17,435 patients with two hospitalizations, 8.1% who had DAMA during their first hospitalization also experienced DAMA during their second hospitalization. In contrast, only 0.8% of patients without DAMA during their first hospitalization experienced DAMA during their second hospitalization.

### 3.3. Factors Associated with Discharge Against Medical Advice

In the multivariable regression model, different variables were associated with an increased risk of DAMA. The ≤50 years (OR: 1.73; 95% CI: 1.30–2.14) and 51–60 years (OR: 1.39; 95% CI: 1.07–1.81) age groups were associated with an increased risk of DAMA compared with the >80 years age group and male sex (OR: 1.46; 95% CI: 1.23–1.72). The strongest associations were observed for cancer types. Compared with urinary tract cancer as the reference group, lip, oral cavity, and pharynx (OR: 4.29; 95% CI: 2.89–6.37), larynx (OR: 4.20; 95% CI: 2.54–6.92), and liver cancer (OR: 4.01; 95% CI: 2.51–6.43) were strongly positively associated with DAMA. Significant positive associations were also evident for the esophagus and stomach, colon and rectum, pancreas, bronchus and lung, skin, female genital organs, and lymphomas. Distant metastases were not associated with a risk of DAMA (Table 2).

## 4. Discussion

This national multicenter cross-sectional study, which analyzed data from hospitalized adult cancer patients in Germany, identified a DAMA prevalence of 0.9%. Compared with the general hospital population, where DAMA rates are reported to range between 1% and 2% [1,2], the lower rates among cancer patients may reflect the structured and intensive follow-up care inherent to oncology treatment. Such care often includes personalized treatment plans and the establishment of robust support networks, which strengthen patient–provider relationships and enhance adherence to recommended protocols [14]. Additionally, the multidisciplinary nature of oncology care, characterized by regular interactions between patients and healthcare teams, likely fosters trust and ensures continuity of care, contributing to reduced DAMA rates. However, although cancer patients seem to exhibit lower DAMA rates overall, certain subgroups, such as younger or male patients, may be disproportionately affected and might require specific attention. The variability observed between demographic and clinical subgroups suggests the need for targeted, patient-centered interventions to address the unique challenges faced by high-risk populations.

Our findings indicate that younger patients exhibit higher rates of DAMA, potentially due to competing responsibilities, such as work and family obligations, which may conflict with the extended hospitalizations often required for oncology treatment. Similarly, a higher DAMA rate was observed among male patients, potentially reflecting societal norms and expectations that prioritize external responsibilities over personal health [15]. In contrast, older patients in our study had lower DAMA rates, which may be due to reduced external obligations, increased awareness of health risks, and physical or cognitive limitations that require greater reliance on healthcare providers [16].

Elevated DAMA rates were observed among patients with cancers strongly associated with smoking, including cancers of the lip, oral cavity, oropharynx, larynx, and lungs. Although this study does not directly establish a causal relationship between smoking and DAMA behavior, the findings are consistent with the existing literature suggesting that patients with substance use disorders, such as nicotine dependence accompanied by withdrawal symptoms and behavioral challenges, may have an increased risk of leaving against medical advice [3]. For instance, Hwang et al. emphasize the significant role of smoking as a risk factor for DAMA [3]. Similarly, our analysis revealed higher DAMA rates among patients with liver cancer, a malignancy frequently linked to chronic alcohol consumption [17]. Patients with alcohol use disorders may be more prone to DAMA due to factors such as withdrawal symptoms, comorbid psychiatric conditions, and an elevated likelihood of non-adherence to hospital regulations [18]. In line with these findings, Kraut et al. conducted a population-based study in Manitoba, Canada, analyzing nearly two million hospital discharges [19]. The authors identified substance use disorders, particularly alcohol and drug abuse, as significant predictors of AMA. The diagnostic group of alcohol and drug abuse exhibited the highest AMA rate (11.71%). Socioeconomic disadvantages, such as lower household incomes, and comorbid conditions, including HIV/AIDS, were also strongly associated with higher AMA rates. Importantly, Kraut et al. demonstrated that a history of AMA was the strongest predictor of subsequent AMA events [19]. Similarly, our analysis revealed that patients who experienced DAMA during a prior hospitalization were more likely to exhibit DAMA during subsequent hospitalizations. Among 17,435 patients with two hospitalizations, 8.1% of those with DAMA during their first hospitalization also left against medical advice during their second hospitalization, compared with only 0.8% of those without DAMA in their first hospitalization. These findings highlight the cyclical nature of DAMA behavior and underscore the importance of targeted interventions, such as inpatient detoxification programs and counseling, as demonstrated by Kuo et al., to address the complex interplay between substance use and psychosocial challenges [20].

In addition, the elevated rates of DAMA observed in patients with certain types of cancer suggest that these patients may face unique challenges or perceive their prognosis differently than other cancer patients. For example, cancers of the lip, oral cavity, and pharynx are more prevalent among individuals with lower socioeconomic status, which may exacerbate the physical and psychological distress of these patients and increase their likelihood of DAMA [21,22]. Addressing these disparities requires both patient-level interventions and systemic health policy changes to ensure equitable access to psychosocial and socioeconomic support.

Conversely, the lowest DAMA rates in our study were observed among patients with breast, prostate, and urinary tract cancers. These findings may reflect the well-established follow-up care and robust support systems typically available to these patient groups. For instance, breast cancer patients often benefit from extensive advocacy networks, specialized care services, and structured continuity of care, all of which likely enhance adherence to treatment plans and reduce the likelihood of DAMA [23]. Expanding similar systems of care to other cancer subgroups with higher DAMA rates may contribute to reducing disparities and improving outcomes across oncology populations.

Furthermore, the lack of a significant association between the presence of distant metastases and DAMA rates found in the present study may support the hypothesis that non-clinical factors, such as psychological, social, and economic challenges, may have a greater impact on DAMA decisions than disease severity alone.

Overall, the findings of the present study indicate the potential importance of addressing non-clinical determinants, such as psychosocial distress, substance use, and socioeconomic barriers, within the context of oncology care. While this study did not directly evaluate specific interventions, the results suggest that focusing on these factors may help reduce DAMA rates, particularly among high-risk groups such as younger patients, male patients, and those with cancers associated with higher DAMA rates. Tailored educational programs and personalized follow-up plans that address patients’ individual needs and barriers to adherence may be promising strategies. Improving patient–provider communication may also play a key role. Approaches such as motivational interviewing, which focus on patient-centered communication and addressing ambivalence about treatment, may support informed decision making and adherence [24]. In addition, early integration of psycho-oncology support and palliative care may help manage emotional distress, such as anxiety and depression, which are often associated with more burdensome cancers and may influence DAMA decisions [25]. These approaches warrant further investigation to determine their potential effectiveness in reducing DAMA rates and improving treatment adherence.

Substance use, which is potentially a significant driver of DAMA in certain patient populations, warrants focused and targeted interventions. Comprehensive smoking cessation programs and support for patients with alcohol-related problems, including structured detoxification and counseling, have shown promise in reducing DAMA rates [20]. Addressing these challenges early in the care pathway may play an important role in mitigating their negative impact on treatment adherence. Such interventions are particularly relevant in oncology care, where the complexities of cancer treatment may exacerbate the risks associated with untreated substance use disorders [17].

Reducing socioeconomic barriers also appears to be a critical component of improving care adherence and addressing DAMA. Practical support measures, such as transportation assistance, childcare services, and financial counseling, might help alleviate the external pressures that often lead patients to prematurely leave the hospital. These approaches could reduce obstacles disproportionately affecting vulnerable patient populations, enabling them to focus on their treatment. Flexible hospital policies, such as short-term outpatient care options or day treatment programs, might also provide patients with the flexibility to manage external responsibilities without compromising adherence to treatment plans [18].

Healthcare professionals, particularly clinical nurse specialists (CNSs) and advanced nurse practitioners (ANPs), could play a pivotal role in implementing strategies to address discharge against medical advice (DAMA). CNSs, with their specialized expertise in oncology care, have been shown to effectively manage issues such as malnutrition through patient education and nutritional management during treatment [26]. These interventions not only improve patients’ physical health but may also enhance their ability to adhere to treatment plans. Similarly, ANPs, with their advanced clinical training and focus on personalized care, contribute by fostering effective communication between patients and providers, alleviating psychological distress, and supporting adherence to prescribed treatments [27].

Together, CNSs and ANPs are well-positioned to address the multifactorial drivers of DAMA by promoting continuity of care and integrating psychosocial and palliative care services into oncology practice [27,28]. Their involvement in addiction management is particularly relevant for patients with smoking- or alcohol-related cancers, where targeted interventions may significantly reduce DAMA risks [20]. Integrating these specialized professionals into oncology care teams could enhance the delivery of holistic, patient-centered care, addressing the complex interplay of psychosocial, behavioral, and socioeconomic factors contributing to DAMA.

Future research is needed to evaluate the impact of these interventions on DAMA rates and to explore additional strategies for addressing the unique needs of high-risk cancer populations. Such efforts could inform the development of tailored, evidence-based approaches aimed at reducing DAMA and improving both treatment adherence and patient outcomes in oncology care. By systematically addressing the underlying determinants of DAMA, oncology care frameworks may better support vulnerable patient populations, ultimately contributing to improved health outcomes and reduced disparities in cancer treatment.

### Strengths and Limitations

This study has several limitations that must be considered when interpreting the findings. First, the cross-sectional design limited the ability to infer causality between the observed associations of demographic, clinical, and psychosocial factors with DAMA rates. Second, the reliance on administrative hospital data may have introduced potential misclassification or the omission of critical variables, such as comprehensive socioeconomic data or detailed information on substance use, including smoking and alcohol consumption. These omissions limited insights into lifestyle factors that may influence DAMA behavior and provide valuable context for understanding the observed patterns. However, while smoking and substance use were not directly analyzed, cancer types strongly associated with smoking and alcohol consumption may indirectly suggest a link between these behaviors and DAMA decisions.

Additionally, the anonymized nature of the dataset restricted the ability to link hospitalization data with outpatient follow-up records, constraining the capacity to assess long-term outcomes and readmissions after DAMA. Another limitation was the exclusion of mental and behavioral health diagnoses (ICD F00–F99), which are often critical determinants of DAMA decisions. This exclusion may have prevented valuable insights into the influence of psychiatric conditions. Furthermore, the reliance on ICD coding limited the ability to account for variability in treatments, such as major surgeries versus minor interventions, which may also influence DAMA rates. While this study did not capture individual tumor stages, it did investigate the association with metastases, which are often linked to advanced tumor stages. This analysis provides at least indirect insights into the role of disease progression in DAMA decisions.

Despite these limitations, this study has notable strengths. The large, multicenter national dataset from diverse hospitals across Germany enhances the generalizability of the findings. Furthermore, this study provides novel insights into DAMA among cancer patients, an understudied yet vulnerable population. By identifying key demographic and clinical predictors of DAMA, this study lays a foundation for future research and the development of patient-centered interventions aimed at reducing DAMA rates, improving adherence to care, and enhancing outcomes for oncology patients.

## 5. Conclusions

This study provides novel insights into the factors associated with DAMA in cancer patients, highlighting the potential role of demographic and behavioral characteristics. The analysis did not identify a significant association between DAMA and the presence of distant metastases, suggesting that clinical severity may not be a primary driver. Instead, factors such as younger age, male sex, and cancers often linked to smoking and alcohol use were observed more frequently in patients who left against medical advice, suggesting that non-clinical influences may play a central role.

Although DAMA rates in cancer patients were lower than those observed in the general hospital population, the identification of significant subgroup differences highlights the need for further research into the underlying determinants of DAMA in this vulnerable group. This study contributes to the existing literature by focusing on cancer patients and provides a foundation for future investigation. Further research is needed to validate these findings and explore targeted interventions that address both clinical and non-clinical factors influencing DAMA. Such efforts could improve adherence and outcomes for cancer patients.

## Figures and Tables

**Table 1:** Baseline characteristics of study sample.

Variable	Hospitalizations (*n* = 88,246)
Mean age (standard deviation)	66.5 (13.8)
≤50	10,358 (11.7)
51–60	16,216 (18.4)
61–70	24,609 (27.9)
71–80	22,976 (26.0)
>80	14,087 (16.0)
Female	47,333 (53.6)
Male	40,913 (46.4)
Length of stay in hospital in days (median, IQR)	4 (7)
Distant metastases	25,951 (29.4)
Cancer type	
Lip, oral cavity, and pharynx	4770 (5.4)
Esophagus and stomach	4318 (4.9)
Colon and rectum	8679 (9.8)
Liver	2055 (2.3)
Pancreas	3170 (3.6)
Larynx	1482 (1.7)
Bronchus and lung	9506 (10.8)
Skin	5403 (6.1)
Female breast	10,965 (12.4)
Female genital organs	4790 (5.4)
Prostate	6219 (7.0)
Urinary tract	8121 (9.2)
Lymphomas	5990 (6.8)
Leukemias	2564 (2.9)
Secondary diagnoses	
Diabetes mellitus	13,502 (15.3)
Lipid metabolism disorders	8896 (10.1)
Hypertension	37,439 (42.4)
Coronary heart disease	6825 (7.8)
Atrial fibrillation and flutter	7790 (8.8)
Chronic kidney disease	5709 (6.5)
Thyroid gland disorders	10,282 (11.7)

**Table 2:** Association of demographic and clinical variables with discharge against medical advice in patients hospitalized for cancer (multivariable logistic regression).

Variable	OR (95% CI) *	*p*-Value
≤50 years	1.73 (1.30–2.14)	<0.001
51–60 years	1.39 (1.07–1.81)	0.014
61–70 years	1.21 (0.94–1.55)	0.133
71–80 years	0.96 (0.74–1.24)	0.748
>80 years	Reference	
Female	Reference	
Male	1.46 (1.23–1.72)	<0.001
Distant metastases	0.96 (081–1.13)	0.594
Cancer type		
Lip, oral cavity, and pharynx	4.29 (2.89–6.37)	0.060
Esophagus and stomach	2.78 (1.80–4.29)	<0.001
Colon and rectum	2.73 (1.84–4.05)	<0.001
Liver	4.01 (2.51–6.43)	<0.001
Pancreas	2.45 (1.49–4.03)	<0.001
Larynx	4.20 (2.54–6.92)	<0.001
Bronchus and lung	2.80 (1.89–4.17)	<0.001
Skin	2.53 (1.65–3.89)	<0.001
Female breast	0.60 (0.35–1.02)	<0.001
Female genital organs	2.34 (1.46–3.76)	<0.001
Prostate	1.06 (0.65–1.74)	0.812
Urinary tract	Reference	
Lymphomas	1.85 (1.19–2.88)	0.006
Leukemias	1.61 (0.93–2.79)	0.092
Co-diagnoses		
Diabetes mellitus	1.00 (0.63–0.87)	0.980
Lipid metabolism disorders	0.96 (0.73–1.25)	0.742
Hypertension	0.74 (0.63–0.87)	<0.001
Coronary heart disease	0.75 (0.55–1.03)	0.078
Atrial fibrillation and flutter	0.89 (0.66–1.19)	0.419
Chronic kidney disease	1.21 (0.91–1.63)	0.196
Thyroid gland disorders	0.89 (0.70–1.15)	0.381

* Multivariable logistic regression adjusted for age, sex, cancer type, co-diagnosis, and metastases.

## Data Availability

The data that support the findings of this study are available from the corresponding author upon reasonable request.

## Supplementary Materials

The following supporting information can be downloaded at: https://www.mdpi.com/article/10.3390/cancers17010056/s1, STROBE Statement—checklist of items that should be included in reports of observational studies.
